# Influence of N-Arachidonoyl Dopamine and N-Docosahexaenoyl Dopamine on the Expression of Neurotrophic Factors in Neuronal Differentiated Cultures of Human Induced Pluripotent Stem Cells under Conditions of Oxidative Stress

**DOI:** 10.3390/antiox11010142

**Published:** 2022-01-10

**Authors:** Ekaterina Novosadova, Oleg Dolotov, Ludmila Inozemtseva, Ludmila Novosadova, Stanislav Antonov, Darya Shimchenko, Vladimir Bezuglov, Anna Vetchinova, Vyacheslav Tarantul, Igor Grivennikov, Sergey Illarioshkin

**Affiliations:** 1 Institute of Molecular Genetics, National Research Centre Kurchatov Institute, 123182 Moscow, Russia; novek-img@mail.ru (E.N.); olegd@img.ras.ru (O.D.); lsi@img.ras.ru (L.I.); novosadova-l@rambler.ru (L.N.); vamore@inbox.ru (S.A.); shimchenko1211@mail.ru (D.S.); tarantul@img.ras.ru (V.T.); igorag@img.ras.ru (I.G.); 2 Faculty of Biology, Lomonosov Moscow State University, Leninskie Gory, 119234 Moscow, Russia; 3 Shemyakin-Ovchinnikov Institute of Bioorganic Chemistry of RAS, 117997 Moscow, Russia; vvbez2013@yandex.ru; 4 Research Center of Neurology, 125367 Moscow, Russia; annvet@mail.ru

**Keywords:** oxidative stress, hydrogen peroxide, induced pluripotent stem cells, neurotrophic factors, dopaminergic neurons, endocannabinoids, N-acyl dopamines

## Abstract

Oxidative stress (OS) is implicated in the pathogenesis of several neurodegenerative diseases. We have previously shown that N-acyl dopamines (N-ADA and N-DDA) protect the neural cells of healthy donors and patients with Parkinson’s disease from OS. In this study, we assessed the effects of N-acyl dopamines on the expression of neurotrophic factors in human-induced pluripotent stem cell-derived neuronal cultures enriched with dopaminergic neurons under conditions of OS induced by hydrogen peroxide. We showed that hydrogen peroxide treatment increased BDNF but not GDNF mRNA levels, while it did not affect the secretion of corresponding proteins into the culture medium of these cells. Application of N-acyl dopamines promoted BDNF release into the culture medium. Under conditions of OS, N-DDA also increased TRKB, TRKC and RET mRNA levels. Furthermore, N-acyl dopamines prevented cell death 24 h after OS induction and promoted the expression of antioxidant enzymes GPX1, GPX7, SOD1, SOD2 and CAT, as well as reduced the BAX/BCL2 mRNA ratio. These findings indicate that stimulation of the expression of neurotrophic factors and their receptors may underlie the neuroprotective effects of N-acyl dopamines in human neurons.

## 1. Introduction

N-acyldophamines are endogenous conjugates of dopamine and long-chain fatty acids. N-docosahexaenoyl dopamine (N-DDA) and N-arachidonoyl dopamine (N-ADA), as well as some other N-acyldopamines, exhibited potency to activate cannabinoid receptor-1 (CB1) and vanilloid TRPV-1 receptor [1,2] and thus can be attributed to the large family of cannabinoid-like/endovanilloid compounds [3].

These compounds have previously been shown to have antioxidant and neuroprotective properties in various models of oxidative stress (OS) in vitro and in vivo. Bobrov et al. demonstrated the antioxidant activity of N-ADA using a galvinoxyl test that allows calculation of the stochiometric number (SN). SN corresponds to the number of active hydroxyl groups of the tested compound in the reduction of galvinoxyl radical molecules. The SN value of N-ADA was close to 2, while lipid antioxidant (+)α-tocopherol reduced galvinoxyl with SN close to 1. Using primary cultures of rat cerebellar granule neurons (CGN) in various models of OS (hydrogen peroxide, K+/serum deprivation and glutamate exposure), the authors demonstrated the neuroprotective effect of N-ADA, which was realized via reducing elevated hydroperoxide levels within the cells by a mechanism that did not involve activation of cannabinoid or vanilloid receptors [4]. N-DDA also demonstrated a comparable N-ADA SN value in the galvinoxyl test (2.01). Moreover, single administration of N-DDA (10 mg/kg) after 10-day treatment with neurotoxin MPTP in the animal model of Parkinson’s disease increased locomotor activity of animals 1.8 times [5]. Grabiec et al. used organotypic rat hippocampal slice cultures to investigate the neuroprotective effect of N-ADA application under conditions of OS evoked by N-methyl-D-aspartate (NMDA) excitotoxicity. It was demonstrated that a concentration of 1 nM N-ADA protected dentate gyrus granule cells and caused a slight reduction in the number of microglial cells. This effect was blocked only by CB1-receptor inverse agonist/antagonist AM251 and was insensitive to CB2, TRPV1 and TRPA1 receptor antagonists [6].

The involvement of the CB1 receptor in the neuroprotective properties of N-ADA was also demonstrated by Vedunova et al. in a model of hypoxia-induced changes in spontaneous bioelectric activity of primary culture of mouse hippocampal neurons. N-ADA application (10 μM) prevented changes in the activity of neuronal network observed in the control on day 1 after hypoxia. The authors found that the blockade of CB1 and TRPV1 receptors reduced the protective effect of N-ADA on day 7 [7]. Therefore, the neuroprotection mechanism of N-ADA may include signaling via the TRPV1 receptor.

In the model of acute neonatal hypoxia (ANH) in rat pups, Sukhanova et al. showed that treatment with N-ADA significantly attenuated the delayed development of sensorimotor reflexes and stress-evoked disruption of memory retention in hypoxic rats but had no effect on hypoxia-induced hyperactivity. In rats exposed to hypoxia, treatment with N-ADA decreased GPx2 gene expression and increased the GSH/GSSG ratio in whole brains 1.5 h after ANH. The authors suggest that the decreased antioxidant enzyme expression and the increased GSH/GSSG ratio after post-hypoxic N-ADA administration could be indicators of decreased oxidative stress in brain structures essential for cognitive and emotional functions [8]. N-ADA protection of neuronal cells under hypoxic conditions may be explained in part by inducing the transcription of genes involved in the hypoxic response. Soler-Torronteras et al. studied the hypoximimetic activity of N-ADA in the human neuroblastoma cell line SKN-SH subjected to hypoxia (1% O_2_) for 3 h and showed that N-ADA led to hypoxia inducible factor-1α (HIF-1α) stabilization, and this effect was independent of CB1 and TRPV1 receptor activation [9].

Recently, we have shown that N-ADA and N-DDA exert neuroprotective effects in the cultures of neuronal progenitors differentiated from human induced pluripotent stem cells (iPSC) obtained from a healthy donor in the model of OS induced by hydrogen peroxide [10]. Further experiments performed on neuronal progenitors and cultures of terminally differentiated neurons enriched with dopaminergic (DA) neurons obtained from iPSC of healthy donors, as well as from patients with Parkinson’s disease, confirmed the neuroprotective effect of these compounds in the same model of OS [11]. Moreover, the protective effect of N-ADA and N-DDA was not associated with their interaction with hydrogen peroxide. It was suggested that neuroprotective and antioxidant effects of these compounds could be mediated through the induction of expression of a number of neurotrophic and antiapoptotic factors in these cells [11]. It is well known that neurotrophic factors (NTFs) play a key role in the development, differentiation, synaptogenesis and survival of brain neurons and in the processes of their adaptation to external influences, including acute or chronic stress [12]. Within a wide class of NTFs, the family of neurotrophins and the family of glial cell derived neurotrophic factor (GDNF) are distinguished. Among the representatives of the neurotrophin family, NGF, BDNF and NT-3 are the most important compounds for their involvement in the functioning of the nervous system. Specific receptors for mature neurotrophins and their signaling are the proteins of the TRK family possessing tyrosine kinase activity [13].

NGF mainly binds to TrkA and BDNF, NT4 binds to TrkB, and NT3 binds to TrkC and, with less affinity, to TrkA [13]. As a result of interactions with neurotrophins, the Trk receptor dimerizes, autophosphorylates and triggers various signaling cascades, such as PLC and Ras/MAPK (ERK signaling pathway) and PI3K (phosphatidylinositol-3-kinase pathway). Additionally, all neurotrophins interact with the low-affinity non-tyrosine kinase receptor p75NTR [14]. Heterodimerization of sortilin and p75NTR can be induced by binding to the precursors of neurotrophins, such as proBDNF, which can be released by neurons and glial cells and is known to induce apoptosis in neurons [15,16]. Intracellular signaling of GDNF is mediated by its binding to a receptor with tyrosine kinase activity consisting of two subunits, GFRa and Ret [17]. Ret-mediated GDNF signaling includes two main signaling cascades that promote cell survival in various neuronal and non-neuronal populations, Ras/ERK (MARK) and PI3K/Akt pathways [18].

NTF plays an important role in the development of neurodegenerative processes, exerting strong positive effects on the viability and functioning of neurons in Alzheimer’s disease and Parkinson’s disease [19]. One of the critical events in the development of acute and chronic neurodegenerative diseases is the damage and death of neurons due to OS [20]. There is evidence of antioxidant activity and neuroprotective effects of key NTFs, such as BDNF and GDNF [21,22]. However, it is not currently known whether NTFs are involved in the neuroprotective effects of N-acyldophamines under conditions of OS. Therefore, in this work, we investigated the effects of N-ADA and N-DDA on the expression of some key genes of NTF, such as *BDNF*, *GDNF*, *NGF*, *NT3* and their receptors, in the model of OS induced by hydrogen peroxide (H_2_O_2_) in differentiated cultures of neurons (enriched with DA neurons) derived from human iPSC. We also studied the effects of these compounds on the content of BDNF, proBDNF and GDNF proteins in cells and culture medium. In addition, we studied whether the neuroprotective effects of N-acyl dopamines are accompanied by changes in the expression of genes of some antioxidant enzymes, such as GPX1, GPX7, SOD1, SOD2, CAT and apoptosis-related genes *BAX* and *BCL2*.

## 2. Materials and Methods

### 2.1. Chemicals

N-arachidonoyl dopamine (N-ADA) and N-docosahexaenoyl dopamine (N-DDA) were prepared in the Laboratory of Oxylipins of Shemyakin-Ovchinnikov Institute of Bioorganic Chemistry of RAS (Moscow, Russian), as described previously [23]. 

### 2.2. Cell Lines

This work was carried out on the iPSC line (IPSHD1.1S) obtained from skin fibroblasts of a healthy donor using CytoTune ™ -iPS 2.0 Sendai Reprogramming Kit. The reprogramming vector includes the four Yamanaka factors Oct3/4, Sox2, Klf4 and c-Myc, shown to be sufficient for efficient reprogramming [24,25]. The obtained iPSC expressed the essential pattern of specific pluripotency-associated genes, possessed a normal karyotype and were capable of producing the derivatives of three embryonic germ layers [25].

The study complies with the Declaration of Helsinki and was performed approved by the Ethics Committee of the Research Center of Neurology. Written informed consent was obtained from every donor. iPSC lines were cultured in mTeSR medium (“STEM CELL Technologies” Vancouver, BC, Canada) on Matrigel-coated substrates as previously described. The differentiation of iPSCs into neuronal progenitors was performed as previously described [25].

### 2.3. iPSC Culturing and Obtaining Differentiated Neuronal Cell Cultures Enriched by DA Neurons

iPSC propagation and neuronal differentiation were performed as described previously [11,25]. 

#### 2.3.1. Immunofluorescence Staining

Neuronal cells (20,000) were plated in each well of Matrigel-treated 24-well plates and were cultured in DMEM/F12 medium containing 2% serum replacement (“Gibco”, Carlsbad, CA, USA), 1 mM non-essential amino acids (“Paneco”, Moscow, Russia), 2 mM L-glutamine (“ICN Biomedicals Inc.”, Hackensack, NJ, USA), penicillin–streptomycin (50 U/mL; 50 µg/mL) (“Paneco”, Moscow, Russia), 1% B27 supplement (“Life Technologies”, Carlsbad, CA, USA), 5 µM forskolin (“Stemgent”, Cambridge, MA, USA), 20 ng/mL BDNF, 20 ng/mL GDNF and 200 µM ascorbic acid (all from “Peprotech”, Cranbury, NJ, USA) in a CO_2_ incubator at 5% CO_2_ and 37 °C. At day 15, the cultures were fixed with 4% neutral-buffered formaldehyde. Immunofluorescence staining was performed according to a previously described method [25] using β-III-tubulin and tyrosine hydroxylase (TH) antibodies as pan-neuronal and DA neuron-specific markers, respectively. Cells were investigated with an AxioImager Z1 fluorescence microscope equipped with an AxioCam HRM camera using AxioVision 4.8 software (Zeiss, Oberhohen, Germany). The whole surface of each well was imaged for cell counting and the obtained images were analyzed with ImageJ 1.49 software (“NCBI”, Bethesda, MD, USA) using an ITCN plugin (Center for Bio-image Informatics, Santa Barbara, CA, USA).

#### 2.3.2. Estimation of the Neuroprotective Properties of N-ADA and N-DDA in Differentiated Neuronal Cell Cultures Enriched by DA Neurons under Conditions of OS 

Neuronal cells (20,000) were plated in each well of Matrigel-treated 24-well plates and were cultured in DMEM/F12 medium supplemented with 2% serum replacement (“Gibco”, Carlsbad, CA, USA), 1 mM non-essential amino acids (“Paneco”, Moscow, Russia), 2 mM L-glutamine (“ICN Biomedicals Inc.”, Hackensack, NJ, USA), penicillin–streptomycin (50 U/mL; 50 µg/mL) (“Paneco”, Moscow, Russia), 1% B27 supplement (“Life Technologies”, Carlsbad, CA, USA), 5 µM forskolin (“Stemgent”, Cambridge, Massachusetts, USA), 20 ng/mL BDNF, 20 ng/mL GDNF and 200 µM ascorbic acid (all from “Peprotech”, Cranbury, NJ, USA) in a CO_2_ incubator at 5% CO_2_ and 37 °C. On the day of the experiment, the growth medium was changed to DMEM/F12 medium supplemented with 2 mM L-glutamine (“ICN Biomedicals Inc.”, Hackensack, NJ, USA) and penicillin–streptomycin (50 U/mL; 50 µg/mL) (“Paneco”, Moscow, Russia). N-ADA or N-DDA (5 µM) were added to the cells at the same time point and the control cultures were treated with an equal volume of DMSO. Cells were then incubated for 40 min and treated with 200 µM H_2_O_2_; the same volume of growth medium was used for the control wells. At 3 and 24 h time points of incubation, the cells were collected for RNA extraction and preparation of cell lysates and the conditioned growth media were collected for ELISA.

#### 2.3.3. MTT Assay

Neuronal cells were seeded by 20,000 per well and cultured on Matrigel-treated 24-well plates in the DMEM/F12 growth medium. On the day of the experiment, the growth medium was changed to DMEM/F12 medium with 2 mM L-glutamine (“ICN Biomedicals Inc.”, Hackensack, NJ, USA) and penicillin–streptomycin (50 U/mL; 50 µg/mL) (“Paneco”, Moscow, Russia). N-ADA or N-DDA at a concentration of 5 µM was added to the cells at the same time. An equal volume of DMSO was used as the control. To induce oxidative stress 40 min later, the cells were exposed to 200 µM of H_2_O_2_ for 3 and 24 h. In the control group, H_2_O_2_ was omitted. The number of viable cells was measured by the MTT ((3-(4,5-dimethylthiazol-2-yl)-2,5-diphenyltetrazolium bromide)) assay after incubation. The calibration curves were constructed to determine the number of surviving cells. For this purpose, 5, 10, 15, 20, 25, 30, 40, 60 and 80 thousand cells were seeded per well of the 96-well plate. On the next day, the optical densities were measured using the MTT assay, and the calibration curves were constructed, where the optical density values were plotted versus the corresponding cell density.

### 2.4. RNA Isolation and qRT-PCR

Total RNA was extracted from the cells with a TRIzol RNA purification kit (“Invitrogen”, Carlsbad, CA, USA) as recommended by the manufacturer. Then, DNA was removed from the RNA specimens with a DNA-free DNA Removal Kit (“Thermo Fisher”, Waltham, MA, USA). cDNA was synthesized from 2 μg of total RNA using MMLV Reverse Transcriptase (“Evrogen Ltd.”, Moscow, Russia) with random primers. The obtained cDNA was amplified using the CFX96 Touch Real-Time PCR Detection System (“Bio-Rad”, Berkeley, CA, USA). qPCRmix-HS SYBR reaction mixture (“Evrogen Ltd.”, Moscow, Russia) was used. 

### 2.5. Measurement of BDNF, proBDNF and GDNF Proteins with ELISA

Culture media were collected at the indicated time points, centrifuged at 14,000× *g* for 5 min at 4 °C, and the supernatants were then stored at −80° C. For preparation of cell lysates, the cultures were washed three times with cold PBS and treated with 1 mL of 100 mM PIPES lysis buffer, pH 7.0, containing 500 mM NaCl, 2% BSA, 0.2% Triton X-100, 0.1% NaN_3_ and protease inhibitor cocktail (2 μg/mL aprotinin, 2 mM EDTA, 10 μM leupeptin, 1 μM pepstatin and 200 μM PMSF) [26]. After five freeze/thaw cycles, the cell lysates were centrifuged at 14,000× *g* for 5 min at 4 °C, and the supernatants were stored at −80 °C. Total BDNF (mature plus proBDNF), proBDNF and GDNF concentrations in cell supernatants and lysates were determined in triplicate using Human/Mouse BDNF, Human Pro-BDNF and Human GDNF DuoSet ELISA kits (Cat. No: DY248, DY3175 and DY212, respectively; “R&D Systems”, Minneapolis, MN, USA), according to the manufacturer’s instructions.

## 3. Results

In this study, we used human iPSC-derived differentiated neuronal cell cultures enriched by DA neurons. iPSC line of healthy donor was generated by our team previously in the Laboratory of Molecular Genetics of Somatic Cells, Institute of Molecular Genetics of Russian Academy of Sciences (Moscow, Russia).

**Figure 1:**
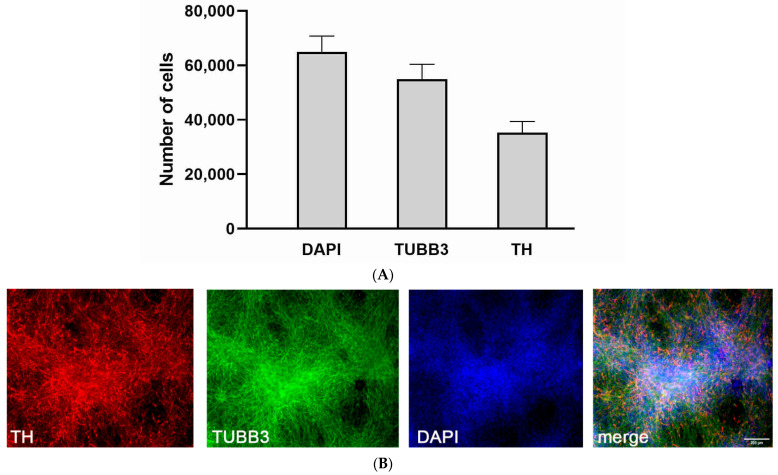
Quantitative characterization and immunofluorescence staining of human iPSC-derived neuronal cell cultures. (**A**). Quantitative characterization of human iPSC-derived neuronal cell cultures. (**B**). Immunofluorescence staining of neuronal cell cultures with β-III-tubulin (TUBB3) (total neuronal population), tyrosine hydroxylase (TH) (DA neurons) antibodies and DAPI (total cell population). The data are represented as mean ± SD, *n* = 7.

According to immunofluorescence staining results, the differentiated cell cultures contained at least 50% DA neurons (Figure 1). 

Earlier, we determined the sensitivity of neuronal cells to H_2_O_2_ and showed that, when using 200 µM of H_2_O_2_, approximately 50% of β-III-tubulin positive cells die [11]. In this way 200 µM H_2_O_2_ was used to induce OS in the investigated cultures. Medium exchange with simultaneous application of N-acyl dopamines N-ADA and N-DDA at a concentration of 5 µM was performed 40 min before H_2_O_2_ treatment. After the addition of H_2_O_2_ 3 and 24 h later, the cells were harvested for the assessment of expression of neurotrophins and their receptors and measurements of BDNF and GDNF proteins in cell lysates and culture medium.

### 3.1. Analysis of Transcription Levels of Neurotrophic Factors and Their Receptors in Differentiated Neuronal Cultures

Using real-time PCR, we assessed the expression of neurotrophic factors *NGF*, *GDNF*, *BDNF*, *NT3* and corresponding receptors *TRKA*, *RET*, *GFRα*, *TRKB95*, *TRKB145*, *TRKC*, *P75* and *SORTILIN*. The obtained results are presented in Table 1.

It was noted that, after 3 h of incubation, H_2_O_2_ decreased the transcription of *BDNF*, *NT3* and *RET* and had no reliable effects on the transcription of other genes. At the same time, both N-acyl dopamines stimulated expression of *BDNF* and *GDNF.* However, only N-DDA was shown to increase the expression of *TRKB95*, *TRKB145*, *NT3*, *TRKC* and *RET*.

Twenty-four hours after OS induction, a decrease in the gene expression for receptors *TRKA*, *RET*, *GFRα*, and increase for *NGF* was observed. In the same conditions but in the presence of N-acyl dopamines, increased expression for *TRKA*, *RET* and *GFRα* was identified. A decrease in *TRKB95* and *P75* expression was observed at the same time. There was also an increase in *NT3* expression after N-DDA, but not N-ADA treatment.

### 3.2. Analysis of BDNF, proBDNF and GDNF Protein Levels in Differentiated Neuronal Cultures by ELISA

**Figure 2:**
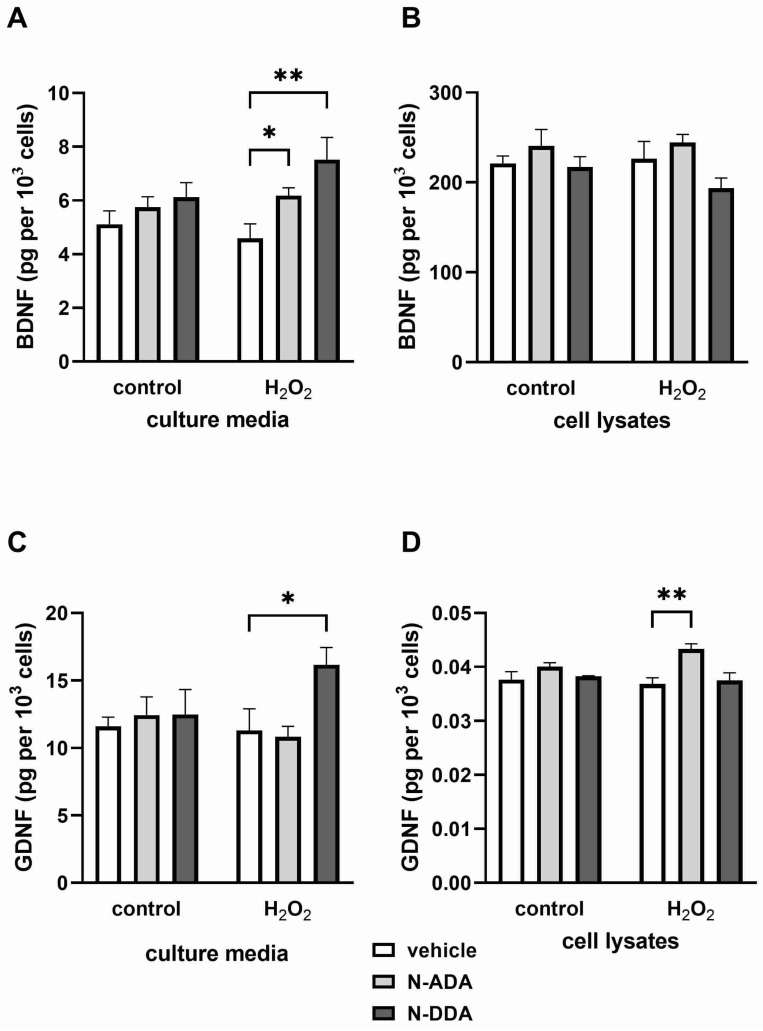
Effects of H_2_O_2_ and N-acyl dopamines on BDNF and GDNF protein levels in the culture medium and cell lysates from human iPSC-derived neuronal cultures 3 h after OS induction. Cell cultures were pretreated for 40 min with N-acyl dopamines (5 µM) and incubated for 3 h with 200 µM H_2_O_2_. BDNF and GDNF levels in cell lysates (**B**,**D**) and culture media (**A**,**C**) were measured by ELISA. (**A**): H_2_O_2_ does not affect BDNF levels in the culture media. N-ADA and N-DDA elevate BDNF levels in the culture media only in H_2_O_2-_treated cells. (**B**): N-acyl dopamines and H_2_O_2_ do not affect intracellular BDNF levels. (**C**): H_2_O_2_ and N-ADA do not affect GDNF levels in the culture media. N-DDA elevates BDNF levels in the culture media only in the presence of H_2_O_2_. (**D**): N-DDA and H_2_O_2_ do not affect GDNF cell content. N-ADA elevates GDNF cell content only in the presence of H_2_O_2_. The data are represented as mean ± SEM (*n* = 3–6). * *p* < 0.05, ** *p* < 0.01.

In the next stage of the work, we analyzed the levels of BDNF and GDNF proteins in culture medium and cell lysates 3 h after H_2_O_2_-induced OS. The obtained results are shown in Figure 2. 

According to the obtained results, both N-ADA and N-DDA promote BDNF release into the culture medium, while the level of BDNF in cell lysates showed a small decrease, which was statistically insignificant. A different profile was observed for GDNF, whose level in lysates significantly increased only after N-ADA application, while in the culture medium, it increased only after N-DDA treatment. Importantly, these data are consistent with our results on the mRNA expression of these neurotrophins. As can be seen from Table 2, the elevated expression of BDNF and GDNF is observed in the presence of both N-acyl dopamines.

**Figure 3:**
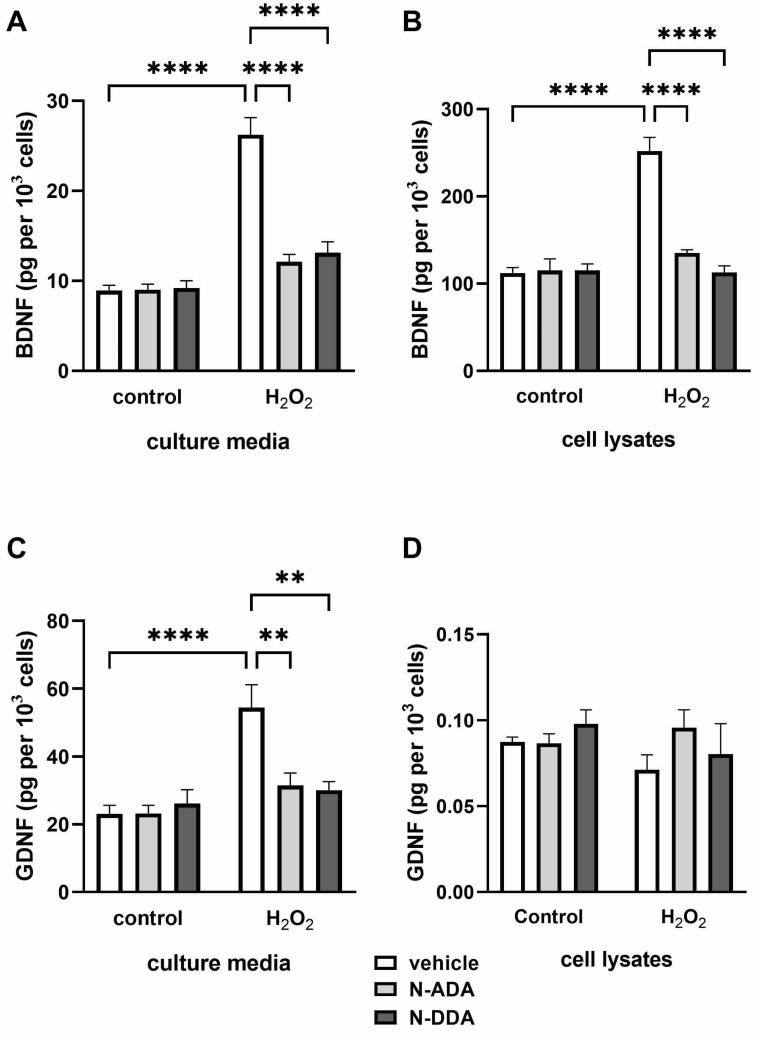
Effects of H_2_O_2_ and N-acyl dopamines on BDNF and GDNF protein levels in the culture medium and cell lysates from human iPSC-derived neuronal cultures 24 h after OS induction. Cell cultures were pretreated with N-acyl dopamines (5 µM) for 40 min and incubated for 24 h with 200 µM H_2_O_2_. BDNF and GDNF levels in the cell lysates (**B**,**D**) and culture media (**A**,**C**) were measured by ELISA. N-ADA and N-DDA prevent the H_2_O_2_-induced increase in BDNF (**A**) and GDNF levels (**C**) in the culture media but do not affect them without H_2_O_2_-treatment. (**B**): H_2_O_2_ elevates the BDNF intracellular level. N-ADA and N-DDA prevent H_2_O_2_-induced elevation in BDNF cell content. (**D**): N-ADA, N-DDA and H_2_O_2_ do not affect GDNF intracellular levels. The data are represented as mean ± SEM (*n* = 3–6). ** *p* < 0.01, **** *p* < 0.0001.

Thus, we have shown that 3 h after OS induction in the presence of N-ADA and N-DDA, the expression of *BDNF* and *GDNF* genes and their release from the cells is stimulated, although N-ADA had a significantly less pronounced effect. Analysis of BDNF and GDNF protein levels in neuronal cultures 24 h after H_2_O_2_ treatment but without the application of N-acyl dopamines, revealed increased BDNF levels in lysates and culture medium, while an increase of GDNF was observed only in the culture medium (Figure 3). When N-acyl dopamines are applied in the presence of H_2_O_2_, BDNF and GDNF levels remain at a level comparable to that in intact cells.

**Figure 4:**
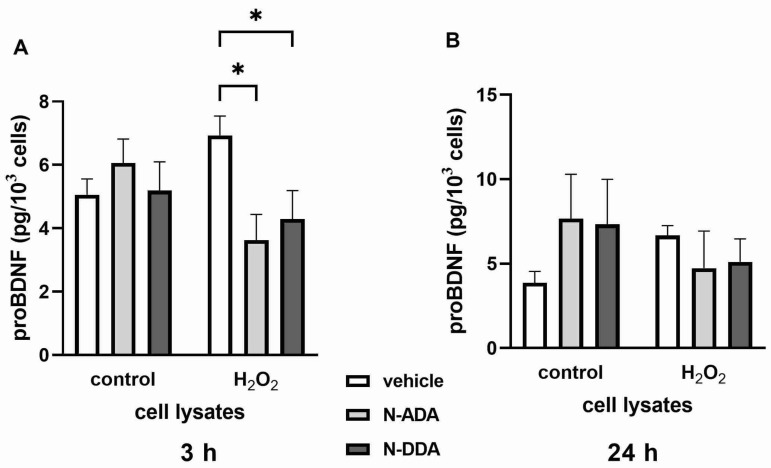
Effects of H_2_O_2_ and N-acyl dopamines on proBDNF protein levels in the cell lysates from human iPSC-derived neuronal cultures 3 h and 24 h after OS induction. Cell cultures were pretreated with N-acyl dopamines (5 µM) for 40 min and incubated with 200 µM H_2_O_2_. ProBDNF levels in the cell lysates were measured by ELISA. (**A**): After 3 h of OS, N-ADA and N-DDA decreased the intracellular proBDNF content in H_2_O_2_-treated cells. (**B**): After 24 h of OS, N-ADA, N-DDA and H_2_O_2_ do not affect proBDNF intracellular content. The data are represented as mean ± SEM (*n* = 3). * *p* < 0.05.

Since a recent report demonstrated that the applied here Human/Mouse BDNF DuoSet ELISA kit cross-reacts 100% with proBDNF [27] and therefore detects total BDNF (mature BDNF plus proBDNF), we additionally measured the effects of N-ADA and N-DDA on the levels of proBDNF under OS conditions using a specific proBDNF ELISA kit. In cell lysates, the predominant form of BDNF is mature BDNF (more than 97%). After 3 h but not 24 h of OS, both N-ADA and N-DDA caused a decrease in intracellular levels of proBDNF (Figure 4). At 3 h and 24 h of OS, the levels of proBDNF in the culture media were found to be below the detection limit of the ELISA kit (78.1 pg/mL, 2.4 pmol/l). Therefore, the detected changes in BDNF levels in the culture media can be attributed to the changes in the levels of mature BDNF.

### 3.3. Assessment of the Effect of N-acyl Dopamines on the Survival of Differentiated Neurons under OS Conditions

**Figure 5:**
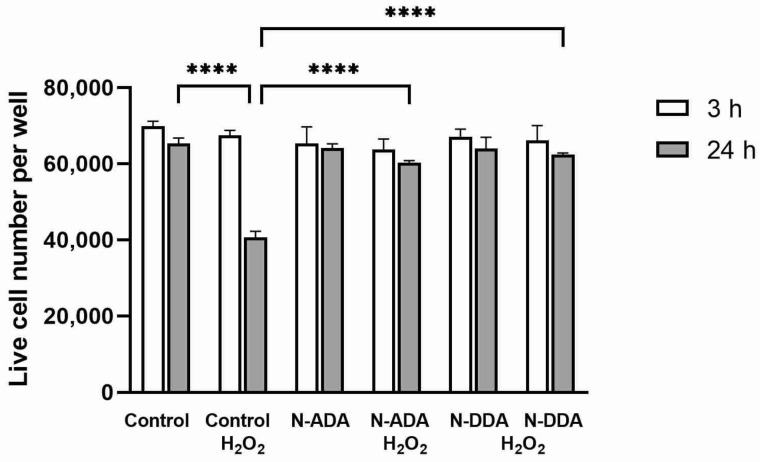
Pretreatment with N-acyl dopamines (5 µM) protects human iPSC-derived TDNs against H_2_O_2_.-induced cell death. Cell cultures were pretreated for 40 min with N-ADA or N-DDA (5 µM) and incubated for 3 h or 24 h with 200 µM H_2_O_2_. Counting of surviving cell numbers was assessed by their detachment with trypsin at indicated time points after H_2_O_2_ administration. The data are represented as mean ± SEM (*n* = 3). **** *p* < 0.0001.

We evaluated the effects of N-acyl dopamines on the survival of differentiated neurons under OS conditions and found that 24 h after the addition of H_2_O_2_, the number of dead cells was about 40%, while there were no statistically significant changes in cell survival in the presence of investigated compounds (Figure 5).

In sum, the obtained results highlight the antioxidant and neuroprotective effects of the investigated compounds.

### 3.4. Analysis of BAX and BCL2 Expression Changes in Differentiated Neuronal Cultures under OS Conditions and Application of N-acyl Dopamines

Next, we analyzed the expression levels of the *BAX* and *BCL2* genes in differentiated neuronal cultures upon the addition of N-acyl dopamines after 3 and 24 h of OS. These results are shown in Table 3.

As can be seen from Table 3, after 3 h of OS, the ratio of *BAX/BCL2* expression in the experimental samples was practically equal to the control. After 24 h of H_2_O_2_ treatment the *BAX/BCL2* expression ratio increased almost 8-fold compared to the control value. Application of N-ADA and N-DDA reduced this ratio 2.2 and 1.5 times, respectively.

### 3.5. Analysis of Changes in the Expression of Several Antioxidant Cell Defense Genes in Differentiated Neuronal Cultures upon N-DDA Application under OS Conditions

We investigated the changes in the expression of several genes encoding proteins of the antioxidant cell defense system 24 h after OS induction in the presence of N-DDA.

**Figure 6:**
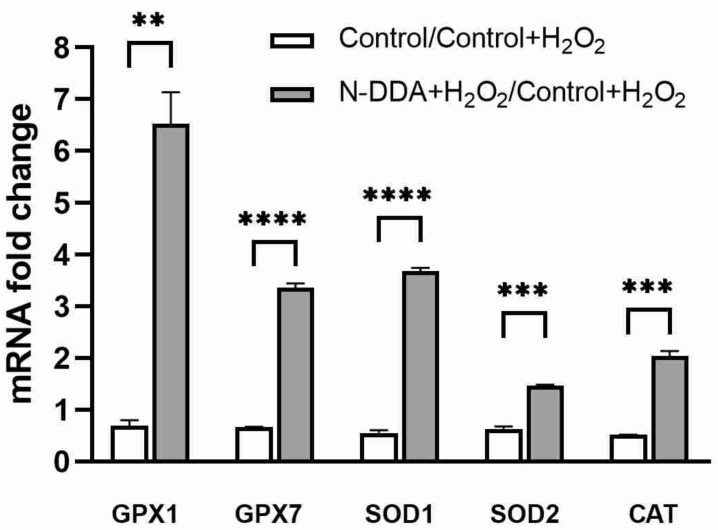
Transcription levels of the genes of some antioxidant enzymes upon N-DDA (5 µM) treatment in differentiated neuronal cultures 24 h of OS induced by 200 µM H_2_O_2_. GPX1: glutathione peroxidase 1, GPX7: glutathione peroxidase 7, SOD1: cytoplasmic superoxide dismutase, SOD2: mitochondrial superoxide dismutase, CAT: catalase. Real-time PCR data, ** *p* < 0.01, *** *p* < 0.001, **** *p* < 0.0001.

As can be seen from the results shown in Figure 6, a significant increase in gene expression of several antioxidant enzymes, GPX1, GPX7, SOD1, SOD2 and CAT, was observed 24 h after the OS in the presence of N-DDA.

## 4. Discussion

The formation of reactive oxygen species (ROS), which are highly reactive, constantly occurs during normal cell metabolism. In pathological conditions, intense ROS generation causes oxidative destruction of proteins, lipids, nucleic acids and carbohydrates. In the body, the toxic effect of ROS is prevented through the functioning of the antioxidant defense system, represented by enzymatic and non-enzymatic components [28]. The cells of the central nervous system are especially sensitive to ROS due to their high metabolic rate, unique lipid composition, and minimal cell content renewal [29]. OS is one of the most significant mechanisms of damage to nervous tissue, which is accompanied by a chain of pathological reactions that irreversibly damage the cell and lead to the triggering of genetically programmed neuronal death, apoptosis [30]. 

The main result of this work is the establishment of a new fact that acute administration of N-acyl dopamines belonging to the family of cannabinoid-like compounds under OS conditions promotes the expression and secretion of key NTFs BDNF and GDNF in differentiated human neuronal cultures enriched with DA neurons. OS induced by H_2_O_2_ (3 h) led to a decrease (2.6 times) in the expression of mRNA for *BDNF*, but not *GDNF*, in our cell cultures (Table 1). After 24 h, we did not find the effect of H_2_O_2_ on the expression of mRNA for BDNF and GDNF but observed significantly increased levels of BDNF and GDNF in the culture medium (Figure 3), which can be explained by the release of NTF from cells into the medium due to cell death in the presence of H_2_O_2_ and in the absence of N-acyl dopamines. The significant increase in the content of BDNF, but not GDNF, observed in cell lysates 24 h after the induction of OS (Figure 3) may indicate a relatively specific stimulation of BDNF protein synthesis by H_2_O_2_. This is consistent with previously published data on the stimulation of H_2_O_2_ at a concentration close to that used by us (250 μM) of mRNA expression for BDNF, but not several other neurotrophic factors, in the culture of PC12 cells that underwent neuronal differentiation [31]. Three hours after induction of OS, we observed a decrease in *NT-3* and *RET* expression, and after 24 h, an increase in the expression of mRNA for *NGF*, and decrease of expression of *TRKA*, the gene encoding its high-affinity receptor, as well as the genes of GDNF receptors *RET* and *GFRα* (Table 1). It remains unclear which cell types are responsible for the increased production of NTF under OS conditions. In healthy adult brain neurotrophins, BDNF and NT-3 are expressed by both neurons and glial cells [32], while GDNF is expressed almost exclusively by neurons, but brain damage induces its expression in glial cells [33]. It was previously demonstrated that moderate damage to DA neurons caused by the H_2_O_2_ treatment (100 μM) increases the expression of *GDNF* mRNA (1 h) and protein (24 h) in astrocytes in mixed neuronal–glial cultures of postnatal rat substantia nigra [34]. It was shown that N-ADA and N-DDA application increased *BDNF* and *GDNF* transcription in the cells 3 h after induction of OS (Table 1). At the same time, N-ADA and N-DDA increased BDNF levels in the culture medium (Figure 2) and decreased the intracellular content of the precursor of BDNF (proBDNF) (Figure 4), suggesting that N-ADA and N-DDA stimulate the release of both mature BDNF and proBDNF from neuronal cells under OS conditions. An alternative interpretation may be that in OS, these N-acyl dopamines promote not only *BDNF* transcription and the release of mature BDNF but also stimulate intracellular BDNF maturation. The levels of proBDNF in the culture media did not exceed the picomolar range of concentrations, while proBDNF induces apoptosis at subnanomolar concentrations [15], suggesting that N-acyl dopamines do not stimulate the release of proBDNF to the apoptosis-inducing levels but promote the release of neuroprotective mature BDNF. Moreover, N-DDA, but not N-ADA, increased GDNF levels in the culture medium of neuronal cells. Additionally, N-DDA, but not N-ADA, increased mRNA levels of full-length and truncated forms of *TRKB*, *NT-3*, its high-affinity *TRKC* receptor, and the *RET GDNF* receptor. After 24 h, both investigated compounds enhanced the OS-induced decrease of *NGF* expression, but increased the expression of *GFRα*, *RET* and slightly of *TRKA*. N-ADA, but not N-DDA, attenuated the expression of the *P75* receptor and the truncated form of the *TRKB* receptor after 24 h of OS conditions (Table 1). In sum, these data indicate the ability of N-ADA and N-DDA to differentially regulate OS-induced activation of the NTF system, which may be one of the possible pathways for the observed neuroprotective effects of these N-acyl dopamines under OS conditions.

The antioxidant system, which is a complex of finely organized components consisting of a number of enzymes and low molecular weight substances, exerts its functions to prevent damage by high concentrations of ROS in the cell [29]. The coordinated activity of enzymatic and non-enzymatic components of this system provides nonspecific resistance of the organism and adaptive capabilities to the effects of various stress factors. The function of antioxidant enzymes is to maintain a steady-state concentration of peroxides and oxygen radicals. The enzymatic component is divided into two groups: (i) enzymes that inactivate the superoxide anion radical—superoxide dismutase (SOD) and ferroxidase (ceruloplasmin), and (ii) enzymes that quench organic and inorganic peroxides—catalase, glutathione-dependent peroxidases and transferases. Although the mechanisms of neuroprotective effects of NTFs under OS conditions are not completely understood, there is some evidence for their capability to modulate the activity of antioxidant enzymes. Thus, it was shown that BDNF and NGF protect neurons against glutamate-induced excitotoxicity suppressing the accumulation of peroxides and increasing the activity of SOD, GPX and CAT in rat hippocampal neuron cultures [35]. Injection of GDNF into the rat hippocampus under kainate-induced excitotoxicity conditions increased the hippocampal activity of SOD and GPX and decreased the generation of free hydroxyl radicals [36]. These data indicate that NTFs can activate the antioxidant enzyme system. We tested whether N-DDA affects the expression levels of several antioxidant enzyme genes. It was found that 24 h after the addition of H_2_O_2_, N-DDA stimulates the expression of genes of the components of the antioxidant defense of cells. Thus, the expression of glutathione peroxidase (GPX1) is increased 6-fold. This enzyme is capable of catalyzing the reduction of various hydroperoxides, thereby protecting cells from oxidative damage. The expression of catalase (CAT) is increased twofold, and the expression of cytoplasmic (SOD1) and mitochondrial (SOD2) superoxide dismutases is increased 3.6 and 1.5 times, respectively (Figure 6). It is known that cell survival at the early stages of the apoptotic cascade primarily depends on the balance between pro- and anti-apoptotic proteins of the BCL2 family, and the ratio of *BAX/BCL2* expression may be a reliable predictor of apoptosis [37]. We analyzed this ratio 3 and 24 h after OS induction with and without the application of N-acyl dopamines (Table 3) and found that 24 h after OS there was a 4-fold increase in the *BAX/BCL2* ratio, which may indicate the initiation of apoptosis in the investigated cells. The application of N-acyl dopamines reduced this value to 2.2 and 1.5 for N-DDA and N-ADA, respectively.

**Figure 7:**
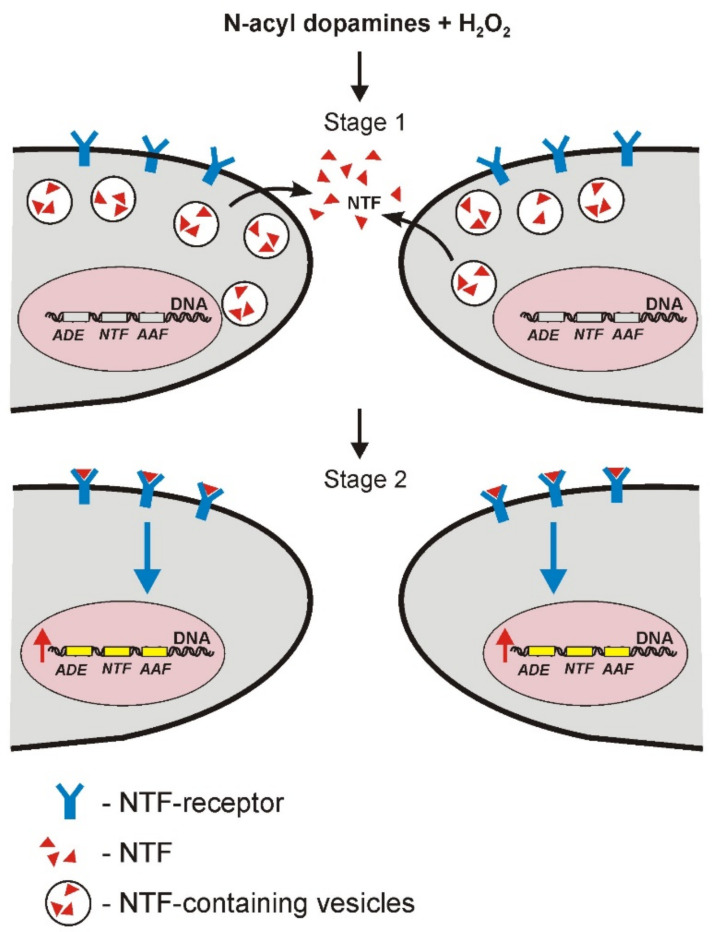
The proposed neuroprotective and antioxidant mechanism of action of N-acyl dopamines in human neurons under OS conditions. Two neuronal cells are presented as an example. The figure schematically shows these effects as a two-stage process. First stage (1) involves the secretion of NTFs (BDNF, GDNF, etc.) by neurons into the culture medium. Second stage (2) consists of binding of these NTF to the surface receptors followed by intracellular signal transduction and activation of gene expression. NTF, neurotrophic factors; ADE, antioxidant enzymes; AAF, antiapoptotic factors. Blue arrow shows signal transduction from NTF receptors to the cell nucleus. Red arrow shows an increase in gene expression.

On the basis of our results, we propose a hypothetical mechanism of neuroprotective and antioxidant action of N-acyl dopamines in human neuronal cultures, which is shown in Figure 7. 

We assume that the action of N-acyl dopamines in the presence of H_2_O_2_ may consist of at least two possible stages. In the first stage, the action of N-acyl dopamines under the oxidative stress condition is associated with NTF (BDNF, GDNF, etc.) secretion by neurons into the culture medium. At the second stage, NTF binding to their corresponding receptors on the surface of neuronal cells takes place, followed by activation of these receptors causing intracellular signal transmission and induction of expression of the genes of corresponding NTF, antioxidant enzymes and anti-apoptotic factors. As a result, products of these genes promote survival and support normal functioning of neurons.

## 5. Conclusions

In this study, we have demonstrated that N-DDA and N-ADA promote cell survival under OS conditions. It was shown for the first time that N-acyl dopamines N-DDA and N-ADA enhance the expression and secretion of such essential NTFs as BDNF and GDNF after 3 h of OS induction by H_2_O_2_. This phenomenon is most likely mediated through promotion of the intracellular pathways that ensure neuronal survival, as no cell death was observed after 24 h of OS upon treatment with the investigated compounds. Thus, our results open up new possibilities for studying the molecular mechanisms of action of N-acyl dopamines at the cellular level under conditions of OS.

## Figures and Tables

**Table 1:** Effects of N-DDA and N-ADA on the expression of NTF and their receptors after 3 and 24 h of OS induction.

Genes	Time	Control/H_2_O_2_	N-ADA + H_2_O_2_/H_2_O_2_	N-DDA + H_2_O_2_/H_2_O_2_
*TRKA*	3 h	1.24 ± 0.63	0.96 ± 0.41	1.8 ± 0.80
	24 h	2.64 ± 0.4 ***	1.62 ± 0.50 *	1.69 ± 0.60 *
*NGF*	3 h	1.66 ± 1.20	1.23 ± 0.29	1.05 ± 0.37
	24 h	0.12 ± 0.01 *	0.59 ± 0.07 *	0.52 ± 0.03 *
*TRKB95*	3 h	3.38 ± 2.3	1.75 ± 0.70	4.00 ± 2.2 *
	24 h	1.92 ± 1.30	0.28 ± 0.04 **	0.85 ± 0.50
*TRKB145*	3 h	1.09 ± 0.36	1.14 ± 0.46	1.62 ± 0.29 *
	24 h	1.90 ± 0.8	1.04 ± 0.03	1.23 ± 0.03
*BDNF*	3 h	2.57 ± 1.29	1.74 ± 0.36 **	3.01 ± 1.2 *
	24 h	1.40 ± 0.7	0.64 ± 0.50	1.05 ± 0.6
*TRKC*	3 h	1.22 ± 0.34	1.10 ± 0.46	1.69 ± 0.49 *
	24 h	2.35 ± 1.0	1.49 ± 0.70	1.33 ± 0.09
*NT3*	3 h	2.17 ± 1.1 *	1.53 ± 0.80	2.32 ± 1.19 *
	24 h	2.23 ± 1.60	0.93 ± 0.60	1.17 ± 0.20
*P75*	3 h	0.85 ± 0.30	0.72 ± 0.06	0.68 ± 0.05
	24 h	1.03 ± 0.30	0.60 ± 0.10 **	0.87 ± 0.40
*SORTILIN*	3 h	1.40 ± 0.59	0.86 ± 0.09	1.58 ± 0.50
	24 h	2.51 ± 1.70	0.96 ± 0.40	1.55 ± 0.60
*RET*	3 h	2.44 ± 1.40 *	1.07 ± 0.44	1.56 ± 0.40 *
	24 h	5.89 ± 1.70 ***	2.68 ± 1.50 *	3.91 ± 0.50 **
*GFR α*	3 h	1.84 ± 0.9	0.71 ± 0.38	1.19 ± 0.35
	24 h	7.90 ± 4.80 *	4.09 ± 2.60 *	3.15 ± 0.51 ***
*GDNF*	3 h	1.57 ± 1.10	1.77 ± 0.40 *	2.32 ± 0.40 *
	24 h	0.85 ± 0.33	1.09 ± 0.40	1.12 ± 0.12

The results of real-time PCR are presented in the form of level ratios of mRNA expression of the different genes in neuronal cultures with (5 µM) and without the addition of N-ADA and N-DDA 3 and 24 h after OS was induced by the addition of 200 µM H_2_O_2_. Data are represented as mean ± SD, *n* = 3–7. * *p* < 0.05, ** *p* < 0.01, *** *p* < 0.001.

**Table 2:** RT-PCR primer pairs.

Gene Name and Accession Number	Direct Primer	Reverse Primer	Annealing T °C
*BAX* NM_138764.5	CGAACTGGACAGTAACATGG	CAGTTTGCTGGCAAAGTAGA	60
*BCL2* NM_000633.3	AGGATTGTGGCCTTCTTTGAGT	CAGAGACAGCCAGGAGAAATCA	60
*GPX7* NM_015696.5	TTGGTCCCATCATTCTTGTGG	GGCTGGTGATTCACTGGTCAA	58
*GPX1* NM_000581.4	TATCGAGAATGTGGCGTCCC	TCTTGGCGTTCTCCTGATGC	62
*SOD1* NM_000454.5	ACTGGTGGTCCATGAAAAAGC	AACGACTTCCAGCGTTTCCT	60
*SOD2* NM_001024465.3	GACAAACCTCAGCCCTAACG	GAAACCAAGCCAACCCCAAC	55
*CAT* NM_001752.4	TAAGACTGACCAGGGCATC	CAAACCTTGGTGAGATCGAA	60
*GDNF* NM_000514.4	TGGGTCTGGGCTATGAAACC	ATGCCTGCCCTACTTTGTCA	60
*GFRα* NM_005264.8	GCCTGTGTGCTCCTATGAAG	CTGGCTGGCAGTTGGTAAA	60
*RET* NM_001355216.1	AGCGGCTCTTCAACCTTCTG	CTCCTCTTAACCATCATCTTCTCC	60
*SORTILIN* NM_002959.7	CTGGGTTTGGCACAATCTTT	CACCTTCCTCCTTGGTCAAA	60
*P75* NM_002507.4	GTGGGACAGAGTCTGGGTGT	AAGGAGGGGAGGTGATAGGA	60
*NT3* NM_001102654.2	TGGTTACTTTTGCCACGATCT	CCTTAACGTCCACCATCTGCT	60
*TRKC* NM_002530.4	AAGCAGCCATGGTTCCAACT	CCTTGATGTTCAACCGCTGC	60
*BDNF* NM_170731.5	TTTGGTTGCATGAAGGCTGC	GCCGAACTTTCTGGTCCTCA	60
*TRKB145* AF508964.1	GTTTCATAAGATCCCACTGGA	TGCTGCTTAGCTGCCTGAGAG	60
*TRKB95* AF400441.1	AGGGCAACCCGCCCACGGAA	GGATCGGTCTGGGGAAAAG	60
*NGF* NM_002506.3	CATACAGGCGGAACCACACT	TTAAACAGCCTGGGGTCCAC	60
*TRKA* NM_001012331.2	TCAACAAATGTGGACGGAGA	GTGGTGAACACAGGCATCAC	60
*18S* KY962518.1	CGGCTACCACATCCAAGGAA	GCTGGAATTACCGCGGCT	60

**Table 3:** Influence of N-acyl dopamines on the ratio of *BAX/BCL2* expression in differentiated neuronal cultures after 3 and 24 h of OS.

Treatment	*BAX/BCL2*	
	3 h	24 h
control	0.66 ± 0.23	0.46 ± 0.21
H_2_O_2_	0.43 ± 0.13	3.81 ± 0.29 *
N-ADA + H_2_O_2_	0.50 ± 0.09	2.26 ± 0.84 *
N-DDA + H_2_O_2_	0.57 ± 0.30	1.56 ± 0.01 **

The results of real-time PCR are given as the ratio of *BAX* to *BCL2* mRNA levels in differentiated neuronal cultures with and without the addition of N-ADA or N-DDA 3 and 24 h after the OS induced by 200 µM H_2_O_2_ treatment. * *p* < 0.05, ** *p* < 0.01.

## Data Availability

Data is contained within the article.
